# What Are the Real Procedural Costs of Bariatric Surgery? A Systematic Literature Review of Published Cost Analyses

**DOI:** 10.1007/s11695-017-2749-8

**Published:** 2017-05-26

**Authors:** Brett Doble, Sarah Wordsworth, Chris A. Rogers, Richard Welbourn, James Byrne, Jane M. Blazeby, By-Band-Sleeve Trial Management Group, Jane Blazeby, Richard Welbourn, James Byrne, Barnaby C. Reeves, Sarah Wordsworth, Robert C. Andrews, Janice L. Thompson, Graziella Mazza, Chris A. Rogers

**Affiliations:** 1 Health Economics Research Centre, Nuffield Department of Population Health, University of Oxford, Oxford, OX3 7LF UK; 2 Clinical Trials and Evaluation Unit, School of Clinical Sciences, University of Bristol, Bristol, BS2 8HW UK; 3 Department of Upper Gastrointestinal and Bariatric Surgery, Musgrove Park Hospital, Taunton, TA1 5DA UK; 4 Southampton University Hospitals NHS Trust, Southampton, SO16 6YD UK; 5 Bristol Centre for Surgical Research, School of Social and Community Medicine, University of Bristol, Bristol, BS8 2PS UK

**Keywords:** Adjustable gastric banding, Costs, Bottom-up costing, Gastric bypass, Gross costing, Micro-costing, Obesity, Sleeve gastrectomy, Top-down costing

## Abstract

This review aims to evaluate the current literature on the procedural costs of bariatric surgery for the treatment of severe obesity. Using a published framework for the conduct of micro-costing studies for surgical interventions, existing cost estimates from the literature are assessed for their accuracy, reliability and comprehensiveness based on their consideration of seven ‘important’ cost components. MEDLINE, PubMed, key journals and reference lists of included studies were searched up to January 2017. Eligible studies had to report per-case, total procedural costs for any type of bariatric surgery broken down into two or more individual cost components. A total of 998 citations were screened, of which 13 studies were included for analysis. Included studies were mainly conducted from a US hospital perspective, assessed either gastric bypass or adjustable gastric banding procedures and considered a range of different cost components. The mean total procedural costs for all included studies was US$14,389 (range, US$7423 to US$33,541). No study considered all of the recommended ‘important’ cost components and estimation methods were poorly reported. The accuracy, reliability and comprehensiveness of the existing cost estimates are, therefore, questionable. There is a need for a comparative cost analysis of the different approaches to bariatric surgery, with the most appropriate costing approach identified to be micro-costing methods. Such an analysis will not only be useful in estimating the relative cost-effectiveness of different surgeries but will also ensure appropriate reimbursement and budgeting by healthcare payers to ensure barriers to access this effective treatment by severely obese patients are minimised.

## Introduction

Bariatric surgery is considered the most efficacious treatment for severe and complex obesity [1] and can be performed using a number of different surgical techniques. In 2013, Roux-en-Y gastric bypass (RYGB) was the most common procedure, followed by sleeve gastrectomy (SG) and adjustable gastric banding (AGB), accounting for 45, 37 and 10% of surgeries performed respectively worldwide [2]. Recently, however, SG has surpassed RYGB as the most common procedure in some jurisdictions, with SG accounting for over 50% of bariatric surgeries performed in the USA [3].

Evidence from retrospective studies suggest that RYGB and SG are likely to result in the greatest amount of weight loss and improvement of comorbidities, but have greater risks and less flexibility than AGB, which is associated with less reliable long-term weight loss [4]. However, evidence from randomised controlled trials comparing the different surgeries is limited [5, 6], as is information on the relative cost-effectiveness of the three surgery types [7]. The lack of the latter evidence could be due partly to a dearth of cost information for the three procedures. Detailed cost estimates (i.e. expenditures involved in acquiring resources that are necessary for the delivery of care) are also a requisite for the development of appropriate reimbursement rates by healthcare payers [8]. For example, the National Health Service (NHS) in the UK has two main tariffs used to reimburse physicians/hospitals for performing bariatric surgery [£4028 (US$5771) for AGB and SG and £4608 (US$6602) for RYGB] [9]. However, the underlying resources and costs attributed to these tariffs are unclear, which could lead to either over- or underpayment of providers of bariatric surgery.

To fill these evidence gaps, a multi-centre randomised controlled trial, known as the By-Band-Sleeve (BBS) study, is being conducted comparing both the relative effectiveness and cost-effectiveness of RYGB, AGB and SG [5, 10]. In comparison to other bariatric surgery trials, the BBS study will assess both clinical and economic outcomes for the three most common approaches to bariatric surgery, in the largest sample size studied in a comparative trial to date (expected to randomise 447 patients per group), over a substantial follow-up period (36 months). However, an important first step in estimating the economic outcomes in the BBS study will be to obtain detailed and ‘accurate’ costs of the three types of bariatric surgery.

The costs associated with bariatric surgery are a function of the resources consumed and the unit costs associated with those resources. These parameters can be identified using a number of different approaches, but can broadly be separated into methods used to identify cost components (gross costing or micro-costing) [11] and methods used to value cost components (top-down costing and bottom-up costing) (see Box 1 for definitions) [12]. The choice of method is usually dependent on the context, data and funding available to conduct a costing study, but it is important to note that the application of different methods may result in variations in the magnitude and therefore the accuracy and reliability of the estimated costs [13, 14]. Detailed cost estimates, derived using transparent methods, are not only important to provide appropriate reimbursement but also act as accurate inputs in analyses comparing the relative cost-effectiveness of the different surgical approaches and will allow healthcare providers to budget judiciously, potentially minimising barriers to access surgery for the increasing number of severely obese patients [15]. Therefore, our study aimed to review the literature and identify robust estimates of the procedural costs of the main types of bariatric surgery. More specifically, we will summarise the international literature on procedural bariatric surgery costs and assess their comprehensiveness, accuracy and reliability.

**Box 1:** Definitions of methods for identifying and valuing cost components

***Methods for identifying cost components*** **Gross-costing:** Involves identifying cost components at a highly aggregated level (e.g. costing an intervention based only on the associated inpatient days) [11].	
**Micro-costing:** A precise method, where an attempt is made to identify every input consumed in the treatment of a particular patient [11]. ***Methods for valuing cost components***	
**Top-down costing:** An approach where relative value units such as hospital days or some other metric are used to separate out relevant costs from comprehensive sources (e.g. the finance department’s annual accounts) and apportion them to individual services or procedures [12]. For example, the sum of the annual budget of an intensive care unit and hospital overhead may be divided by the number of patient days to estimate an average cost per patient per day [34].	
**Bottom-up costing:** An approach where cost components are valued by identifying resource use directly employed for a patient, resulting in patient-specific unit costs [14].	

## Methods

### Eligibility Criteria

Published costing studies in the English language reporting detailed, per-case (i.e. per-patient) cost estimates associated with performing any type of bariatric surgery were considered. To be included, a cost analysis had to report two or more cost components related to performing the procedure. For example, a study reporting a breakdown of the total cost of surgery in terms of inpatient stay, personnel and equipment costs would be included in the review. Studies only reporting the total cost of surgery with no breakdown into individual cost components (i.e. an aggregated cost) were excluded because it is impossible to understand from total costs what underlying resources were included. Studies only reporting detailed total costs for a single component (e.g. equipment costs) were also excluded, even if they broke down the single component into sub-components (e.g. total equipment costs broken down into maintenance costs, operating costs and personnel costs for cleaning). Studies only assessing the cost of healthcare utilisation either before or after bariatric surgery were also excluded.

### Information Sources, Search and Data Collection

Two databases, Ovid MEDLINE and PubMed, were searched from inception to January 16, 2017 (Appendix). Search terms were initially developed for three different categories, costing terminology, types of bariatric surgery and obesity nomenclature, but subsequently refined to increase the sensitivity of the searches. No restrictions were initially placed on the language of the articles to ensure a large number of relevant studies were not published in languages other than English, but any studies not reported in English were excluded from the review during screening. In addition, a hand search of key journals and the quoted references from the included articles was conducted to identify any additional studies. One author (B.D.) screened the titles and abstracts of all the citations identified from the search strategies, reviewed the full-text articles identified after screening and extracted the data from all included studies.

### Data Items

Study design and population, types of bariatric surgery assessed, data collection methods and types of costs (i.e. cost components) included were extracted. Total costs of the procedure were extracted as well as the cost values associated with each individual component. Costs are reported here in the currency and price year originally listed in the included study. When the price year was not reported, it was assumed that the price year would be 1 year earlier than the year in which the study was published. To compare the cost of bariatric surgery between different studies, total procedural costs were also inflated to a 2016 price year. When prices were reported over multiple years, the most recent price year was used to inflate the total cost to make a conservative assumption. In terms of currency conversion for studies conducted in different countries, costs were adjusted for purchasing power parities (PPPs) (to adjust for price differences between countries, rather than just exchange rates, which do not take price differences into account) and converted to 2016 US dollar PPPs [16]. If total costs were reported in US dollars, but had been converted from the country’s currency in which the study was conducted, the total cost estimate was first converted back to the original currency using the exchange rate provided in the article before inflating, adjusting and converting to 2016 US dollars.

### Assessment of Accuracy, Reliability and Comprehensiveness

To provide an indication of the accuracy, reliability and comprehensiveness of the reported cost estimates, the inclusion of ‘important’ cost components based on criteria outlined by Ismail et al. [17] for conducting costing analyses of surgical interventions was assessed. Ismail et al. reviewed costing approaches for robotic surgeries, in general, and assessed 19 studies, three of which related to bariatric surgery [18–20], for their consideration of six criteria with the objective to create and validate a micro-costing methodology that could be used by surgeons and hospital administrators to evaluate the cost of implementing new surgical approaches. As standardised guidance regarding how to conduct a micro-costing is limited [21, 22], the methodology presented by Ismail et al. provides one of the only frameworks for the conduct of micro-costing studies and has specifically been designed for the evaluation of surgical interventions. The consideration of the six criteria presented by Ismail et al. was therefore thought to form a standard by which existing cost analyses could be measured. We have, however, also added an additional criterion (inclusion of overhead costs) to the six originally presented by Ismail et al. [17] as exclusion of such overheads could also affect the accuracy, reliability and comprehensiveness of cost estimates reported in the literature. A study was considered to include one of the ‘important’ cost components when a separate cost value could be identified for that individual component. The seven ‘important’ cost components included:

1. Cost, not charge data used in the analysis;
2. Operating room costs reported separate from hospital admission costs;
3. Medical device costs reported (e.g. endoscopy column, laparoscopic tower);
4. Personnel costs reported (e.g. surgeon, nurse, anaesthesiologist time);
5. Re-usable instrument costs reported (e.g. bowel graspers, surgical scissors);
6. Disposable instrument/consumables costs reported (e.g. needles, disposable staplers); and
7. Overhead costs reported.

Reporting of the methods used to identify cost components (gross and/or micro-costing) and value cost components (top-down and/or bottom-up costing) was appraised. When methods were not specifically reported, we assigned the relevant method based on the reported data collection description for resource items and costs. The inclusion of specific parameters based on standardised formulas [17] in the calculation of ‘important’ cost components (e.g. medical devices, personnel, re-usable and disposable instruments) was also evaluated. The parameters of interest included:

1. Capacity adjustment of personnel costs (e.g. adjusting salary for working days minus paid leave when determining a per-minute personnel cost);
2. Amortisation or depreciation of medical devices (e.g. allocating the acquisition cost of a device over its useful lifespan);
3. Maintenance fees for medical devices (in addition to the amortised acquisition cost);
4. Adjustments for medical devices shared across different procedures (i.e. laparoscopic tower may only be used 50% of the time for bariatric surgery);
5. Sterilisation costs of reusable instruments (e.g. personnel time disinfecting and repackaging reusable instruments); and
6. Disposal costs of consumables (e.g. the waste management costs associated with consumables).

### Synthesis of Results

Syntheses of the extracted data were performed to compare study characteristics and cost estimates across studies as well as combine individual study cost estimates into summary measures of total procedural costs. Important study characteristics and detailed cost estimates from the included studies were summarised in tabular format and used to evaluate the availability of cost estimates for different types of bariatric surgery, the methods used to collect the cost data and the specific cost components included in the analyses. Mean costs and standard deviations (SD) were calculated for all reported cost estimates together, estimates derived only from cost data (as charges do not necessarily reflect the actual cost of the resources consumed to deliver the surgery) and for different types of bariatric surgery. The inclusion of ‘important’ cost components was also synthesised in tabular format.

## Results

### Study Selection

**Fig. 1:**
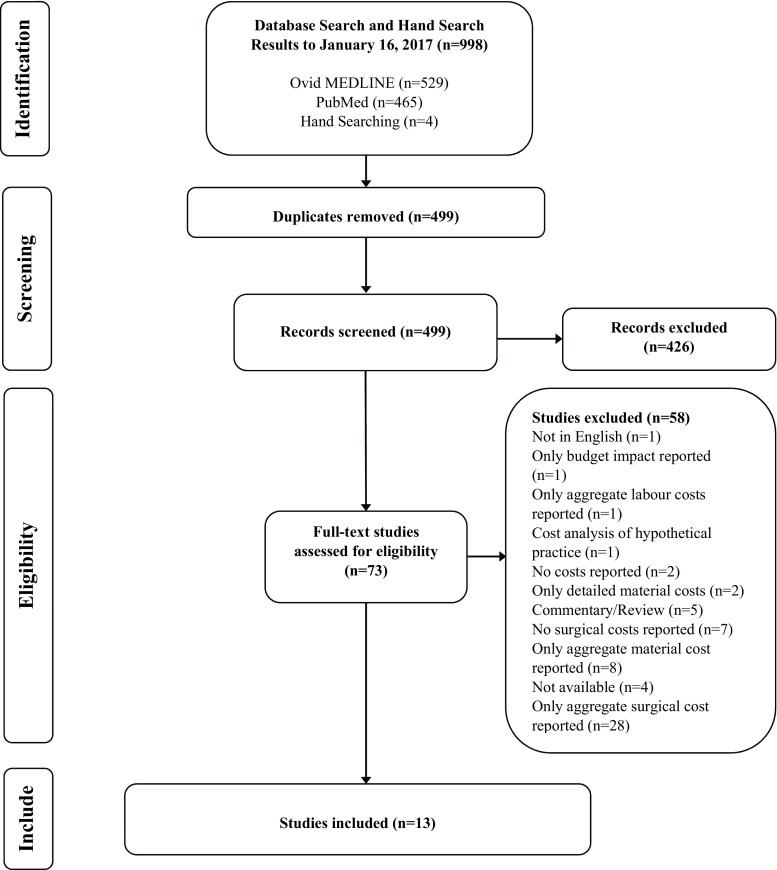
Flow diagram for selection of studies

The search strategy identified a total of 998 citations. After removing duplicates (*n* = 499), 499 unique citations remained for title and abstract screening, which left 73 unique citations of interest for full-text review. After full-text review, 13 studies were selected for detailed review (Fig. 1).

### Study Characteristics

Thirteen studies (Table 1) reported detailed procedural costs [17, 18, 23–33]. The majority of the studies were conducted from a US hospital perspective [23, 26–30, 32, 33], with the remaining studies taking a European [17, 18, 31], Australian [25] and Brazilian [24] hospital perspective. Limited details concerning the sources of data were provided in all the studies. Most commonly, data sources were simply stated as specific hospital departments or general hospital databases [17, 24, 28–30]. Other sources of data included hospital charges or claims data [23, 27, 32, 33], purchase prices [31], a dedicated bariatric surgery database [26], patient case record forms [25] and a mix of prospective, retrospective, documentation and finance department data [18].

**Table 1:** Summary of studies reporting detailed procedural costs for bariatric surgery

Author [ref] currency year^a^	Study design and population	Type(s) of bariatric surgery	Data collection methods	Cost components included	Total procedural cost	Cost component one	Cost component two	Cost component three	Cost component four	Cost component five
Studies using charge (not cost data)										
Cooney [30] USD 2000	June 1998 to March 1999 16 GB procedures performed by single surgeon at Penn State Hershey Medical Center (Pre group) compared to 12 GB procedures performed by same surgeon April to December 1999 (Post group)	Pre-pathway GB (*n* = 16) and post-pathway GB (*n* = 12)	Costs of care obtained from hospital financial information system using cost/charge ratios; OR costs obtained from OR information system	Room and board, OR, supplies, laboratory and radiology and miscellaneous costs; OR time divided into anaesthesia time, patient preparation time, surgical time and ‘wake up’ time	Pre: $10,176 SD $789 Post: $8511 SD $763	Hospital room charge for LOS Pre: $3641 SD $398 Post: $2389 SD $347	Operating room costs Pre: $3467 SD $253 Post: $4251 SD $152	Supply costs during in hospital stay Pre: $1152 SD $194 Post: $679 SD $107	In-house laboratory studies and radiology examinations Pre: $629 SD $84 Post: $312 SD $112	Other miscellaneous costs Pre: $1098 SD $122 Post: $878 SD $179
Muhlmann [27] USD 2002	Case series of 10 robotic-assisted laparoscopic procedures compared to 10 CLP during the learning curve	Robotic and CLP each had: SAGB (*n* = 4), IGS (*n* = 4), revisional bariatric surgery (*n* = 2)	Itemised charges calculated by the institutional billing department; personnel charges, OR time and staff estimated using charge rate per minute based on median time for procedures routinely documented in the OR database	Procedural costs included operation time, special instruments and consumables (details of other costs not reported)	Robotic: $9505 CLP: $6260	Operation time Robotic: $1576 CLP: NR	Special instruments Robotic: $1487 CLP: NR	Consumables Robotic: $182 CLP: NR	NA	NA
Scally [32] USD 2003–2010	Analysis of Medicare claims from 2003 to 2010 for patients who underwent bariatric surgery to determine impact of NCD on costs	Patients (*n* = 72,117) identified using ICD-9 codes and DRG payment codes for bariatric surgery	30-day episode payments abstracted from Medicare claims	Payments for index hospitalisation, re-admissions, physician services, outlier payments and post-discharge ancillary care	Pre-NCD: $14,283 Post-NCD: $14,720	Index admission Pre-NCD: $10,865 Post-NCD: $10,980	Physician services Pre-NCD: $1950 Post-NCD: $2292	NA	NA	NA
Weiner [23] USD 2005	Analysis of BlueCross BlueShield claims data; 29,820 privately insured patients	LB, LGBP, OGBP, other restrictive surgery and unknown	Adjudicated inpatient, outpatient and pharmacy claims on the index date of surgery (e.g. bariatric surgery hospitalisation period or day of outpatient surgery)	Combination of Medicare relative value units and actual charges: inpatient, physician and other services provided in office, pharmacy and non-inpatient services claims	All combined $27,833 LB $22,670 LGBP $28,685 OGBP $28,653 Other $26,592 Unknown $28,391	Inpatient (institution and professional fees) LB $18,622 LGBP $28,237 OGBP $28,096 Other $25,429 Unknown $27,337	Professional office (ambulatory services billed) LB $28 LGBP $170 OGBP $162 Other $301 Unknown $3975	Outpatient and other (imaging, laboratory) LB $194 LGBP $314 OGBP $1050 Other $683 Unknown $46	Pharmacy LB $85 LGBP $82 OGBP $52 Other $70 Unknown $301	NA
Frezza [33] USD 2006	Charges for LGBP and LGB and cost of instruments at the Texas Tech University Health Science Center were evaluated	LGBP (*n* = 93) and LGB (*n* = 27)	Charges and costs were obtained from the hospital	Total charges, hospital and anaesthesiology charges; reusable and disposable instruments	Median total charges LGBP: $10,421 LGB: $10,491	Median hospital charges LGBP: $5787 LGB: $5506	Median anaesthesiology charges LGBP: $1714 LGB: $1369	Reusable instruments (ultrasound, Omni retractor, camera, 45° 5 mm lens) Same cost for both procedures; not clear which one is correct $50,970 or $61,970	Disposable instruments for LGB and LGBP are different, but lists of items are too detailed for summary LGBP: $3516 LGB: $4363	NA
Studies reporting costs for gastric bypass procedures										
Angus [28] USD 2001	Retrospective analyses of 133 patients (59 privately insured and 74 publically insured) at the Nassau University Medical Center from January 2001 to December 2001	LRYGBP (*n* = 11) and open RYGBP	No details provided concerning the collection of cost data other than charts were retrospectively reviewed	Direct (operative and hospital services) and indirect costs	LRYGBP: $6350 SD $75 Open RYGBP: $7894 SD $264	Direct costs (operating room time, operating room supplies, post-anaesthesia care, nursing, pharmaceutical diagnostic and/or therapeutic radiologic studies) LRYGBP: $4180 SD $382 Open RYGBP: $3179 SD $101	Indirect costs (housekeeping, overhead costs, insurance and employee benefits) LRYGBP: $1792 SD $263 Open RYGBP: $2137 SD $285	NA	NA	NA
Nguyen [29] USD 2001	Between May 1999 and March 2001, 155 patients were randomly assigned to undergo either LGBP (*n* = 79) or OGBP (*n* = 76)	LGBP (*n* = 68) and OGBP (*n* = 68)	Costs were derived from the University of California, Davis, Medical Center’s decision support system database	Direct (operative and hospital services costs) and indirect costs	LGBP: $14,087 SD $5237 OGBP: $14,098 SD $8527	Operative costs (operative time and supplies including amortisation of laparoscopic equipment and post-anaesthesia) LGBP: $4922 SD $1927 OGBP: $3591 SD $1000	Hospital service costs (diagnostic, nursing, pharmaceutical, therapeutic and other) LGBP: $2519 SD $1712 OGBP: $3742 SD $3978	Indirect costs (overhead costs including administrative, finance, housekeeping, payroll, insurance and employee benefits) LGBP: $6645 SD $2437 OGBP: $6765 SD $4077	Detailed cost breakdowns of the components of operative (operative time/supplies and post-anaesthesia) and hospital service (diagnostic, nursing, pharmaceutical, therapeutic and other) costs are provided, but have not been summarised here due to space considerations	
Salgado [24] BRL (R$) 2004/2007	Retrospective analysis of direct and indirect costs in 2004 and 2007 at the University Hospital of the Faculty of Medicine of Ribeirao Preto-USP	Roux-en-Y gastric bypass by the standard route (*n* = 9 in 2004 and *n* = 7 in 2007)	Data obtained from the Technical Advisory Office of the Hospital	Hospitalisation, surgery centre, examinations, medications and materials	2004: R$6845 SD R$280 2007: R$7526 SD R$435	Hospitalisation 2004: R$2777 2007: R$2020	Surgery centre (includes anaesthesia) 2004: R$1917 2007: R$3043	Examinations (pre- and postoperative laboratory and imaging) 2004: R$201 2007: R$211	Medications (used before and after surgery) 2004: R$398 2007: R$271	Materials (staplers and catheters) 2004: R$1552 2007: R$1988
Hagen [18] USD 0.91 CHF 2011	Case series of gastric bypass patients at the University Hospital Geneva from June 1997 to July 2010	524 OGBP, 323 LGBP and 143 RGBP patients	Decision analytic model used to model average costs; complications and hospitalisation, OR times collected prospectively; ICU stay captured retrospectively; instrument use documented, material from OR protocols; costs from finance departments	OR materials, postoperative ICU stay, overall hospitalisation and amortisation of the robotic system	OGBP: $23,000 LGBP: $21,697 RGBP: $19,363	ICU stay OGBP: $7144 LGBP: $2143 RGBP: $714	Hospitalisation OGBP: $14,137 LGBP: $14,267 RGBP: $9598	Operating time OGBP: NR LGBP: $3241 RGBP: $4609	OR materials (list of items too detailed for summary) OGBP: $2251 LGBP: $5494 RGBP: $5427	Robotic amortisation purchase price: $1,592,500; yearly maintenance fee 10%; interest rate 5%; duration of use of 7 years
Ismail [17] US$1.3345 = €1 2014	Between January and December 2012 86 RGBP performed at Strasbourg’s University Hospital	RGBP (*n* = 86)	Instrument costs and operative times provided by the IGMISIS; personnel and medical devices’ costs retrieved from Strasbourg’s University Hospital	Medical devices, personnel, re-usables and disposables	$10,734	Medical devices $4320 (amortised purchase price and maintenance)	Personnel $1245 (mean duration per person in OR and total payroll costs; adjusted for effective working hours)	Re-usables $1459 (accounts for sterilisation costs and life span of instrument)	Disposables $3630 (number of units used multiplied by purchase price)	NA
Studies reporting costs for gastric banding procedures										
Van Gemert [31] USD (converted from € no conversion rate provided) 1998	21 morbidly obese patients	VBG (*n* = 21)	Costs were based on real prices	Per-patient performances were counted, including operation, revisional surgery, diagnostic and therapeutic procedures, management of surgical complications, outpatient visits and hospital bed days	$5865	Operations (time of surgeon, resident, anaesthesiologist, nursing staff; supplies, equipment, costs of housing, feeding, administration and management) $2601	Laboratory/radiology $300/$219	Endoscopy $22	Physiotherapy $25	Hospital stay/out-patient visits $2192/$506
Keating [25] AUD 2006	Within-trial surgery costs of 30 patients (note other intervention costs reported in the study were over a 2-year period and therefore only surgery costs are reported)	LAGB	Cost data obtained from a private medical specialist and private hospital; resource use documented on patient case record forms by hospital clinical staff	Specialist medical personnel, hospital personnel, LAGB prosthesis, theatre supplies, non-theatre supplies and other expenses	Mean total surgery costs $8527	Specialist medical personnel (surgeon, surgical assistant, anaesthetist) $3000	Hospital personnel $903	LAGB prosthesis $3264	Theatre supplies, non-theatre supplies and other expenses $1654	NA
Ayloo [26] USD 2006–2009	Retrospective review of a prospectively maintained database between March 2006 and October 2009 from University of Illinois at Chicago	LAGB (*n* = 121) and LESS (*n* = 25)	Data entered into dedicated bariatric database; all procedures performed by one surgeon; depreciation estimated; consumable costs represent costs to replace items	Operative time, consumables and laparoscopic tower depreciation	LAGB: $20,346 SD $2069 LESS: $20,502 SD $1885	Operative time LAGB: $3630 SD $662 LESS: $3793 SD $565	Specialised consumables (band, disposable standard or low profile trocars, sutures and other instruments like harmonic scalpel) LAGB: $15,077 SD $1643 LESS: $14,591 SD $1793	Depreciation of laparoscopic tower equipment; based on 5-year life with 200 cases per year	NA	NA

*AUD* Australian Dollars, *BRL* Brazilian Real, *CHF* Swiss Francs, *CLP* conventional laparoscopic procedures, *DRG* Diagnosis-Related Group, *GB* gastric bypass, *ICD-9* International Classification of Disease, version 9, *ICU* intensive care unit, *IGMISIS* Image-Guided Minimally Invasive Surgical Institute of Strasbourg, *IGS* implantable gastric stimulator, *LAGB* laparoscopic adjustable gastric band, *LB* laparoscopic banding, *LESS* laparoendoscopic single-site, *LGB* laparoscopic gastric banding, *LGBP* laparoscopic gastric bypass, *LRYGBP* laparoscopic Roux-en-Y gastric bypass, *NA* not applicable, *NCD* National Coverage Determination, *NR* not reported, *OGBP* open gastric bypass, *OR* operating room, *RGBP* robotic gastric bypass procedure, *RYGBP* Roux-en-Y gastric bypass, *SAGB* Swedish adjustable gastric band, *SD* standard deviation, *VGB* vertical banded gastroplasty

^a^In studies where the price year has not been reported, it was assumed that the price year would be 1 year earlier than the year in which the study was published

A number of different types of bariatric surgery were costed, but no study assessed the cost of the SG procedure, perhaps as this is a relatively new procedure. One study reported costs for bariatric surgery, in general [32], two studies reported costs for different gastric banding procedures [25, 31] and two studies reported costs for gastric bypass procedures [17, 24]. The remaining studies compared the costs of two or more different procedures [18, 23, 26–30, 33].

Most studies included hospitalisation costs [18, 23, 24, 30–33] and/or the costs of consumables/materials/supplies [17, 18, 24–27, 30, 33]. Five studies reported costs associated with operating room time [18, 24, 26, 27, 30], personnel/staff costs [17, 23, 25, 29, 32] (note that only three of these studies [23, 25, 32] explicitly stated that surgeon/physician fees were included in personnel/staff costs) and/or the costs of additional procedures [23, 24, 29–31]. Four studies reported costs associated with a special instrument or technology [17, 18, 26, 27]. Three studies reported the cost of medications [23, 24, 29] and/or a combined cost associated with the operating room, including operating room time, supplies, personnel, equipment, medications and examinations [28, 29, 31]. A few studies also reported intensive care unit/post-anaesthesia costs [18, 29], indirect costs (e.g. overhead, housekeeping, administrative costs, etc.) [28, 29], anaesthesiology costs [33] and other miscellaneous costs [30].

Twenty-three total procedural cost estimates were reported in the 13 included studies, with a mean of $14,389 SD $6110 (range, $7423 to $33,541) (Table 2). Excluding the five studies that used charge data to estimate total procedural costs left 14 estimates with a mean of $13,993 SD $5441. Five studies reported ten estimates of total procedural costs for different types of gastric bypass procedures with a mean of $13,496 SD $4171. Three studies reported four estimates of total procedural costs for different types of gastric banding procedures, with a mean of $15,237 SD $8556.

**Table 2:** Total procedural costs of bariatric surgery reported in 2016 US dollars

Author [ref] currency year	Type(s) of bariatric surgery			Original total cost estimate			Inflated, adjusted, 2016 USD total costs estimate		
Studies using charge (not cost data)									
Cooney [30] USD 2000	Pre-pathway GB	Post-pathway GB		$10,176	$8511		$13,468	$11,265	
Muhlmann [27] USD 2002	Robotic	CLP		$9505	$6260		$12,390	$8160	
Weiner [23] USD 2005	All types combined			$27,833			$33,541		
Frezza [33] USD 2006	LGBP	LGB		$10,421	$10,491		$12,184	$12,265	
Scally [32] USD 2010	Pre-NCD	Post-NCD		$14,283	$14,720		$15,642	$16,120	
Studies reporting total costs for gastric bypass procedures									
Angus [28] USD 2001	LRYGBP	Open RYGBP		$6350	$7894		$8405	$10,567	
Nguyen [29] USD 2001	LGBP	OGBP		$14,087	$14,098		$18,645	$18,659	
Salgado [24] BRL (R$) 2004/2007	RYGB in 2004	RYGB in 2007		R$6845	R$7526		$8359	$9191	
Hagen [18] US$ = 0.91 CHF 2011	OGBP	LGBP	RGBP	₣20,930	₣19,744	₣17,620	$15,575	$14,692	$13,112
Ismail [17] US$1.3345 = €1 2014	RGBP			€14,325			$17,751		
Studies reporting total costs for gastric banding procedures									
Van Gemert [31] USD (from € no rate provided) 1998	VGB			$5865			$8244		
Keating [25] AUD 2006	LAGB			AU$8527			$7423		
Ayloo [26] USD 2009	LAGB	LESS		$20,346	$20,502		$22,554	$22,727	
Mean total cost estimate (all studies) (*n* = 23)							$14,389 SD $6110		
Mean Total Cost Estimate (Only cost data) (*n* = 14)							$13,993 SD $5441		
Mean total cost estimate for gastric bypass procedures (only cost data) (*n* = 10)							$13,496 SD $4171		
Mean total cost estimate for gastric banding procedures (only cost data) (*n* = 4)							$15,237 SD $8556		

*AU$* Australian Dollars, *BRL* Brazilian Real, *CHF* Swiss Francs, *CLP* conventional laparoscopic procedures, *GB* gastric bypass, *LAGB* laparoscopic adjustable gastric band, *LB* laparoscopic banding, *LESS* laparoendoscopic single-site, *LGB* laparoscopic gastric banding, *LGBP* laparoscopic gastric bypass, *LRYGBP* laparoscopic Roux-en-Y gastric bypass, *NCD* National Coverage Determination, *OGBP* open gastric bypass, *RGBP* robotic gastric bypass procedure, *RYGBP* Roux-en-Y gastric bypass, *SD* standard deviation, *VGB* vertical banded gastroplasty

### Inclusion of ‘Important’ Cost Components

The majority of studies used cost [17, 18, 24–26, 28, 29, 31] rather than charge data [23, 27, 32] in their analyses, with two studies [30, 33] using both (Table 3). All the studies using cost data reported operating room costs separate from hospital admission costs, while only two [27, 30] of the four studies using charge data reported these two costs separately. The remaining five ‘important’ cost components (medical device, personnel, re-usable instruments, disposable instruments and overhead costs) varied in their reporting. Nine studies reported costs for instruments [17, 18, 24–27, 29, 30, 33], but usually did not differentiate between re-usable and disposable [18, 24, 25, 29, 30]. Only four studies [17, 18, 25, 29] included five or more of the seven important cost components in their analyses.

**Table 3:** Inclusion of ‘important’ cost components in cost analyses of bariatric surgery

Study	Costs not charges assessed	OR costs separate from hospital admission costs	Medical device^a^ costs reported	Personnel costs reported	Re-usable instrument^b^ costs reported	Disposable instrument^c^ costs reported	Overhead costs reported
Cooney [30]	Charges and costs	Yes	No	No	Yes, but not clear if re-usable or disposable		No
Muhlmann [27]	Charges	Yes	Yes	No	Yes	Yes	No
Weiner [23]	Charges	No	No	No	No	No	No
Frezza [33]	Charges (costs for re-usable and disposable equipment)	No	Yes	No	Yes	Yes	No
Scally [32]	Charges	No	No	Yes	No	No	No
Angus [28]	Costs	Yes	No	No	No	No	Yes
Nguyen [29]	Costs	Yes	Yes	Yes	Yes, but not clear if re-usable or disposable		Yes
Salgado [24]	Costs	Yes	No	No	Yes, but not clear if re-usable or disposable		No
Hagen [18]	Costs	Yes	Yes	No	Yes, but not clear if re-usable or disposable		No
Ismail [17]	Costs	Yes	Yes	Yes	Yes	Yes	No
Van Gemert [31]	Costs	Yes	No	No	No	No	No
Keating [25]	Costs	Yes	No	Yes	Yes, but not clear if re-usable or disposable		No
Ayloo [26]	Costs	Yes	Yes	No	No	Yes	No
Totals	Costs—8 Charges—3 Both—2	Yes—10 No—3	Yes—6 No—7	Yes—4 No—9	Yes—3 Yes, but NS—5 No—5	Yes—4 Yes, but NS—5 No—4	Yes—2 No—11

*NS* not separate, *OR* operating room

^a^Includes items such as endoscopy column, laparoscopic tower, anaesthetic machine, monitors, robotic system (if applicable) etc.

^b^Includes items such as bowel graspers, needle drivers, surgical scissors, forceps etc.

^c^Includes items such as drapes, tip covers, cannula seals, needles, disposable staplers/recharges, gloves, syringe etc.

### Methods and Parameters Used for Calculating ‘Important’ Cost Components

Only one study made specific reference to a method for identifying cost components [17]. Micro-costing methods were assumed to be most commonly employed to identify cost components, but methods of valuing components were not discernible in the majority of the studies. The inclusion of specific parameters in the calculation of ‘important’ cost components was also poorly reported. Only three studies amortised the cost [17, 18] or accounted for depreciation [26] of medical devices over their life span and only two studies accounted for the cost of maintenance fees [17, 18]. One of the studies reporting a combined operating room cost also noted that the laparoscopic equipment costs were amortised [29]. Only one study reported capacity adjusted personnel costs and accounted for sterilisation costs of reusable instruments [17]. No studies made adjustments for medical devices shared across different procedures or included the disposal costs associated with consumables.

## Discussion

This paper presented a systematic review of cost analyses of a number of different approaches to bariatric surgery for the treatment of severe obesity. From the 13 studies included in the review, sources and methods of data collection were minimally reported, making it difficult to ascertain what methods were used to identify and value cost components. A number of different types of bariatric surgery were costed mainly from a US hospital perspective, including laparoscopic gastric bypass and gastric banding procedures, but no study reported a cost for the SG procedure. Some of the reported cost estimates were, however, for open procedures, procedures not commonly performed (e.g. vertical banded gastroplasty) or surgical techniques likely to be limited to certain providers (e.g. robot-assisted surgeries). This limits the generalisability and usefulness of the reported cost estimates for decision-making purposes as the majority of bariatric surgeries, worldwide, are performed laparoscopically using either the RYGB or SG procedure [2].

The inclusion of individual cost components in the total cost estimates varied widely, although the majority of the studies included hospitalisation and consumables/material/supply costs. Interestingly, only three studies explicitly considered surgeon/physician fees, despite the potential for these costs to drive differences in total procedural costs between the three procedures. Consideration of surgeon/physician fees in any future analyses will therefore be important, especially for comparing cost estimates between RYGB and SG where equipment/instrument costs could be quite similar. The variation in included cost components can potentially be explained by the lack of a clearly defined care cycle or timeframe in which resource use and costs were measured for the majority of studies (only two studies defined a care cycle [23, 30]), making it difficult to determine what cost components should be included.

Mean total procedural costs ranged from US$13,307 to US$15,237 depending on the types of studies included in the calculation. Excluding studies using charge data resulted in a lower mean total procedural cost (US$13,993 vs. US$14,389) and mean total procedural costs were observed to be lower for gastric bypass compared to gastric banding procedures (US$13,496 vs. US$15,237). These differences should, however, be interpreted with slight caution as these mean estimates are based on a small number of studies conducted in a number of different countries/settings and for a range of different procedures (e.g., open, laparoscopic and robot-assisted). In comparison to the UK tariffs for bariatric surgery, the mean estimates reported in the literature are much larger [e.g. £4608 (US$6602) for ‘Stomach bypass procedures for obesity—HRG code FZ84Z’ and £4,028 (US$5,771) for ‘Restrictive stomach procedures for obesity—HRG code FZ85Z’]. The basis of the UK tariffs is, however, unknown and their use as a relevant indicator of the accuracy of the costs of bariatric surgery reported in our review may not be appropriate.

Overall, most studies in the review did not report accurate, reliable and comprehensive estimates of the total procedural costs as the inclusion of the ‘important’ costs components based on recommended costing methods for surgical procedures was poor, with no study including all of the components. Calculation methods were also poorly reported and usually did not account for recommended parameters when estimating costs. This is not surprising as detailed cost information for interventions is lacking in many clinical areas.

Systematic reviews of costing studies of bariatric surgery are limited in the published literature. One relevant study was identified that reviewed cost approaches for robotic surgeries, in general [17], and despite the limited overlap in included studies, our review came to a similar conclusion, namely that costing studies related to surgical procedures (or specifically bariatric surgery) have not reported their methods transparently and largely do not consider ‘important’ cost components and parameters required for their estimation.

Furthermore, we have attempted to extract details of the reporting of specific parameters that would be required to generate appropriate cost estimates according to the formulas presented by Ismail et al. [17]. Just because a cost component has been included in an analysis does not mean that parameters required to estimate accurate, reliable and comprehensive costs have been considered. This point is highlighted in our review as even when important cost components were included, the methods and parameters used in their calculation were infrequently reported. Our review has also identified some additional criteria that should be reported to help improve the quality of cost estimates for bariatric surgery. Transparent reporting of the methods of identifying and valuing cost components should be provided, as the choice of method can have an impact on the magnitude of the cost estimates [13, 14]. An explicit care cycle definition should also be provided, especially to differentiate between cost estimates that have and have not included costs that are incurred before and/or after the actual conduct of the procedure, such as the costs of nutritional and psychological evaluations, 6 to 12 months of medical weight management, re-admissions, postoperative complications, routine vitamin supplements and laboratory testing for the life of the patient after surgery. These additional costs may be significant, but from the available literature, it is not clear if such costs were considered due to the lack of care cycle definitions.

Our study does, however, have limitations. First, detailed costings of bariatric surgery may have been conducted by certain healthcare providers, but not available in the literature. This is possibly due to the inclusion of sensitive pricing information (e.g. discounts negotiated with manufacturers of certain equipment) and, therefore, unlikely to be publically available.

Furthermore, our review only identified a small number of studies, with the majority conducted in a US hospital context. This makes it difficult to generalise the results of our study to other settings/jurisdictions, as different equipment, materials and personnel may be involved across different sites and countries. Caution should, therefore, be made when interpreting the mean cost estimates presented in our review in a local context.

Overall, our review indicates that there is a need for up-to-date costings of the three most common bariatric procedures (RYGB, AGB and SG). To ensure these costs are collected in a consistent manner, micro-costing methods have been identified as the most appropriate approach. We plan to conduct such a micro-costing study across a number of hospitals offering bariatric surgery within the NHS in England as part of the BBS study [5, 10]. Our study has been designed to consider all the ‘important’ cost components outlined in this review, will report the parameters involved in their calculation in a transparent manner and explicitly define the cycle of care to ensure inclusion of all relevant cost components. We will, therefore, be able to determine accurate, reliable and comprehensive estimates of the cost of bariatric surgery.

## Appendix

### SEARCH STRATEGY FOR TARGETED LITERATURE REVIEW

**Database:** Epub Ahead of Print, In-process and Other Non-Indexed Citations, Ovid MEDLINER(R) 1946 to Present.

**Date of search:** 22/11/2016

1. exp. “costs and cost analysis”/ or exp health care costs/ or exp health expenditures/ or exp hospital costs/
2. bottom-up.ab,hw,kf,kw,ot,sh,ti,tw.
3. 1 and 2
4. (microcost$ or micro-cost$).ab,hw,kf,kw,ot,sh,ti,tw.
5. (bottom-up adj5 cost$).ab,hw,kf,kw,ot,sh,ti,tw.
6. (bottom-up adj5 accounting).ab,hw,kf,kw,ot,sh,ti,tw.
7. (activity-based adj5 accounting).ab,hw,kf,kw,ot,sh,ti,tw.
8. (activity-based adj5 cost$).ab,hw,kf,kw,ot,sh,ti,tw.
9. 3 or 4 or 5 or 6 or 7
10. time study.ab,hw,kf,kw,ot,sh,ti,tw.
11. time motion study.ab,hw,kf,kw,ot,sh,ti,tw.
12. (time and motion method).ab,hw,kf,kw,ot,sh,ti,tw.
13. time-and-motion method.ab,hw,kf,kw,ot,sh,ti,tw.
14. (time and motion study).ab,hw,kf,kw,ot,sh,ti,tw.
15. time-and-motion study.ab,hw,kf,kw,ot,sh,ti,tw.
16. time motion analysis.ab,hw,kf,kw,ot,sh,ti,tw.
17. 9 or 10 or 11 or 12 or 13 or 14 or 15 or 16
18. exp bariatric surgery/ or exp gastric bypass/ or exp jejunoileal bypass/ or exp anastomosis, roux-en-y/ or exp gastroenterostomy/ or exp gastrostomy/
19. gastric bypass.ab,hw,kf,kw,ot,sh,ti,tw.
20. adjustable gastric banding.ab,hw,kf,kw,ot,sh,ti,tw.
21. gastric banding.ab,hw,kf,kw,ot,sh,ti,tw.
22. sleeve gastrectomy.ab,hw,kf,kw,ot,sh,ti,tw.
23. (Roux-en-Y adj5 gastric bypass).ab,hw,kf,kw,ot,sh,ti,tw.
24. bariatric surgery.ab,hw,kf,kw,ot,sh,ti,tw.
25. metabolic surgery.ab,hw,kf,kw,ot,sh,ti,tw.
26. weight-loss surgery.ab,hw,kf,kw,ot,sh,ti,tw.
27. digestive surgery.ab,hw,kf,kw,ot,sh,ti,tw.
28. 18 or 19 or 20 or 21 or 22 or 23 or 24 or 25 or 26 or 27
29. exp obesity/ or exp obesity, morbid/
30. (obesity adj5 severe).ab,hw,kf,kw,ot,sh,ti,tw.
31. (obesity adj5 complex).ab,hw,kf,kw,ot,sh,ti,tw.
32. morbid obes$.ab,hw,kf,kw,ot,sh,ti,tw.
33. weight-loss.ab,hw,kf,kw,ot,sh,ti,tw.
34. 29 or 30 or 31 or 32 or 33
35. 9 and 28—**Total hits 1**
36. 9 or 17
37. 28 and 36—**Total hits 2**
38. 1 and 28 and 34—**Total hits 327**
39. 1 and 28—**Total hits 499**
40. 1 and 28—**Update 26/12/2016 Total hits 16**
41. 1 and 28—**Update 02/01/2017 Total hits 3**
42. 1 and 28—**Update 09/01/2017 Total hits 8**
43. 1 and 28—**Update 16/01/2017 Total hits 3**

**Database:** PubMed.

**Date of search:** 22/11/2016

1. ((((((costs and cost analysis[MeSH Terms])) OR costs, cost analysis[MeSH Terms]) OR costs, health care[MeSH Terms]) OR costs, hospital[MeSH Terms]) OR costs, treatment[MeSH Terms]) OR health expenditures[MeSH Terms]
2. bottom-up[Title/Abstract]
3. 1 and 2
4. (microcosting[Title/Abstract]) OR micro-costing[Title/Abstract]
5. bottom-up costing[Title/Abstract]
6. bottom-up accounting[Title/Abstract]
7. activity-based accounting[Title/Abstract]
8. activity-based costing[Title/Abstract]
9. 3 or 4 or 5 or 6 or 7 or 8
10. (time and motion studies[MeSH Terms])
11. time study[Title/Abstract]
12. time motion study[Title/Abstract]
13. time-and-motion method[Title/Abstract]
14. time-and-motion study[Title/Abstract]
15. time motion analysis[Title/Abstract]
16. 10 or 11 or 12 or 13 or 14 or 15
17. ((((((((((bariatric surgery[MeSH Terms]) OR surgeries, bariatric[MeSH Terms]) OR gastric bypass[MeSH Terms]) OR gastric bypass, greenville[MeSH Terms]) OR gastric bypass, roux en y[MeSH Terms]) OR bypass, jejunoileal[MeSH Terms]) OR anastomosis, roux en y[MeSH Terms]) OR diversion, roux en y[MeSH Terms]) OR loop, roux en y[MeSH Terms]) OR gastroenterostomy[MeSH Terms]) OR gastrostomy[MeSH Terms]
18. gastric bypass[Title/Abstract]
19. adjustable gastric banding[Title/Abstract]
20. gastric banding[Title/Abstract]
21. sleeve gastrectomy[Title/Abstract]
22. roux-en-y gastric bypass[Title/Abstract]
23. bariatric surgery[Title/Abstract]
24. metabolic surgery[Title/Abstract]
25. weight-loss surgery[Title/Abstract]
26. digestive surgery[Title/Abstract]
27. 17 or 18 or 19 or 20 or 21 or 22 or 23 or 24 or 25 or 26
28. (obesity[MeSH Terms]) OR morbid obesity[MeSH Terms]
29. severe obesity[Title/Abstract]
30. complex obesity[Title/Abstract]
31. morbid obesity[Title/Abstract]
32. weight-loss[Title/Abstract]
33. 28 or 29 or 30 or 31 or 32
34. 9 and 27—**Total hits 1**
35. 9 or 16
36. 27 and 35—**Total hits 4**
37. 1 and 27 and 33—**Total hits 297**
38. 1 and 27—**Total hits 459**
39. 1 and 27—**Update 19/12/2016 Total hits 5**
40. 1 and 27—**Update 26/12/2016 Total hits 1**
